# How News May Affect Markets’ Complex Structure: The Case of Cambridge Analytica

**DOI:** 10.3390/e20100765

**Published:** 2018-10-06

**Authors:** Antonio Peruzzi, Fabiana Zollo, Walter Quattrociocchi, Antonio Scala

**Affiliations:** 1 The Department of Environmental Sciences, Informatics and Statistics (DAIS), Ca’ Foscari University of Venice, Venezia 30123, Italy; 2 Center for the Humanities and Social Change, Venezia 30123, Italy; 3 Istituto dei Sistemi Complessi (ISC)-CNR, UOS Sapienza, Roma 00185, Italy; 4 LIMS London Institute of Mathematical Sciences, London W1K 2XF, UK

**Keywords:** social media, complex networks, financial series

## Abstract

The claim of Cambridge Analytica, a political consulting firm, that it was possible to influence voting behavior by using data mined from the social platform Facebook created a sudden fear in its users of being manipulated; consequently, even the market price of the social platform was shocked.We propose a case study analyzing the effect of this data scandal not only on Facebook stock price, but also on the whole stock market. To such a scope, we consider 15-minutes prices and returns of the set of the NASDAQ-100 components before and after the Cambridge Analytica case. We analyze correlations and Mutual Information among components finding that assets become more correlated and their Mutual Information grows higher. We also observe that correlation and Mutual Information are mutually increasing and seem to follow a master curve. Hence, the market appears more fragile after the Cambridge Analytica event. In fact, as it is well-known in finance, an increase in the average value of correlations augments the systemic risk (i.e., all the market can collapse as a whole) and decreases the possibility of allocating a safe investment portfolio.

## 1. Introduction

Social media platforms like Facebook (FB) have become the main communication medium; however, the concentration of users’ data in the hands of a few big players like FB and Google has raised concerns about the possibility of getting a monopolistic control of information.

In this scenario, the Cambridge Analytica (CA) scandal, brought to the fore in 17 March 2018, has ignited a strong debate. CA was a British political consulting firm that claimed to offer, during the electoral processes, services of strategic communication based on data mining, data brokerage, and data analysis techniques. CA’s role in political campaigns has been controversial and it is still a subject of ongoing criminal investigations; however, the effectiveness of CA’s methods for targeting voters is strongly questioned by political scientists.

The collection of personally identifiable information of at least 87 million Facebook (FB) users collected by CA since 2014 brought up a data scandal, since CA held that those data were allegedly used to attempt to influence voting [1]. Even if FB banned CA and restricted the access to its own data from external companies, the sudden fear that FB data could be used to influence and manipulate people created a shock in the FB stock price.

The impact of event-related news on financial markets has always received privileged attention in academic literature since Eugene Fama conducted his semi-strong tests on the Efficient Market Hypothesis [2,3,4,5,6]. Many of those works focused on the market reaction to common public announcements, such as dividend issues and stock splits. This allowed mitigating the effect of spurious events, but restricted the investigation to a limited number of cases.

The results of event studies making use of intra-day data seem to suggest that the release of new information is quickly reflected in stock returns and in their volatility [7,8]. Moreover, higher volatility seems to persist for several hours following news release [7]. This seems to be true also in the case of intra-day, fixed-income rates and foreign-exchange future rates [9].

Not only news content, but also media coverage might play a relevant role. What seems to emerge is that trading activity and volatility in a company’s stock do increase as the company captures the attention of the media [10,11,12]. In this sense, stale news also seems to influence the behavior of investors [13].

It is also worth noting that particularly relevant and resonating events might trigger periods of market turmoil [14]. In those periods, the correlation of all the stocks in the market seems to increase and, thus, achieving diversification might become difficult [15,16,17]. To this respect, Zheng et al. find that the first Principal Component of assets’ cross-correlation might be used as an effective measure of systemic risk [18].

Eventually, assets’ cross-correlation is not the only dependency measure affected in periods of financial distress. Wang and Hui show, for worldwide market indexes, how their Mutual Information, measuring non-linear dependency, reached a peak in the middle of the 2008–2009 financial crisis [19].

The contribution of this work is to present a case study concerning the impact of a media resonating data scandal (CA) not only on the asset directly involved in the scandal (FB), but also on the whole market. In order to do so, we consider a dataset containing the time series of 15-minute intraday prices of the NASDAQ-100 components spanning from 1 March to 12 April 2018. In Section 2, we analyze volatility, cross-correlations, and Mutual Information of the NASDAQ-100 components. We show how the market becomes more interconnected, and hence more fragile, after the CA event. We discuss the limits and mark the perspectives of our findings in Section 3, considering also possible future developments. Finally, in Section 4, we describe the dataset in detail and recap the methods and models applied in the analysis.

## 2. Results

**Figure 1:**
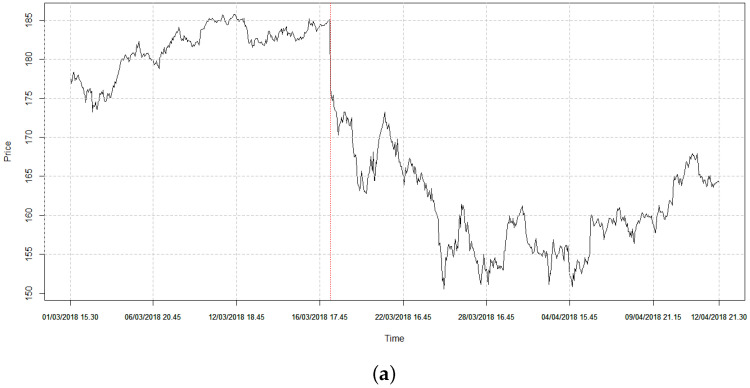

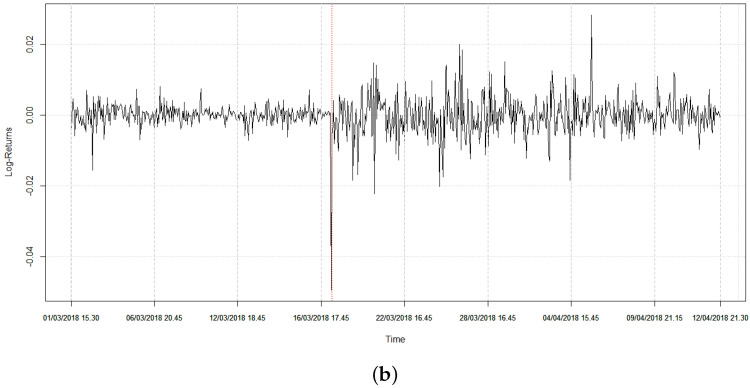
Bloomberg intraday time series for the Facebook (FB) stock. (**a**) Time series for the price. (**b**) Time series for the log-returns. The Cambridge Analytica (CA) event happens at time 313 (the first observation on March 19th, 2018 in the price chart); notice that after the CA event, the price of the stock decreases and its volatility increases.

To explore the impact of the CA event on the market, we first analyze the effect of the event on the most involved stock, i.e., Facebook. In Figure 1, we show both price and log-returns of the FB stock in a period centered on the CA event.

It is clear that not only FB’s price drops down on a lower level, but also that its volatility (i.e., the size of the fluctuations of the log-returns) increases. After the CA scandal, we observe a ∼165% increase in FB volatility and a ∼15% increase in the average volatility for all the assets considered.

We also consider the 10 stocks showing the highest values of volatility before and after CA. In Table 1, we notice the presence of FB among the 10 stocks showing the highest volatility after CA. There seems to be also an increase in the number of technology-related stocks, from 3 to 6. This may suggest that the shock had an impact not only on FB, but also on technology-related stocks.

Volatility could be detrimental for investors since it increases risks and associated costs; a powerful tool to reduce risks in fluctuating markets is the application of portfolio techniques [20] that rely on correlation to reduce the volatility of investments associated with a set of stocks, i.e., *the portfolio*. However, if the whole market becomes more correlated, the possibility of *systemic* failures appears [21] as the market becomes more fragile.

### 2.1. Correlations

**Figure 2:**
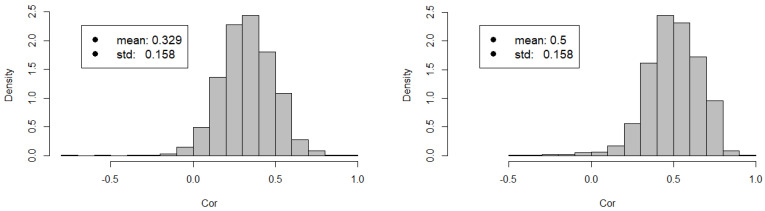
Distribution of cross-correlation among the time series of the NASDAQ-100 components. **Left panel:** Cross-correlation of the time series *before* the CA event. **Right panel:** Cross-correlation of the time series *after* the CA event. Cross-correlations have been computed on two samples of 15-minute intraday stock returns consisting of 311 observations each. Notice that the average correlation of the stock market experiences a ∼50% increase.

**Figure 3:**
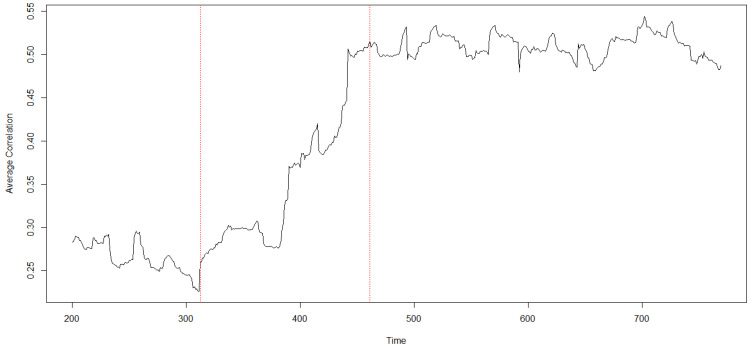
Average cross-correlation among the time series of the NASDAQ-100 components calculated with a moving window of 150 intraday observations. The dashed vertical red lines indicate a window centered around the CA event. Notice the sharp rise of correlations experienced by the stock market around the CA event.

To understand whether the CA event has impacted the whole NASDAQ-100, we analyze correlations among the stocks. To this respect, related methodologies are presented in Section 4.2.1. In Figure 2, we show the histograms for stocks’ correlations before and after the CA event. We observe that while the qualitative shape and the standard deviation of the probability distribution function remain the same, the whole market becomes more correlated since it experiences a ∼50% increase in the average value of cross-correlations. To confirm such observation, we perform a moving average analysis of the cross-correlations. In Figure 3, we show that average cross-correlations are stationary before and after the CA event, shifting from correlations $\langle \rho_{xy}\rangle ∼0.3$ before to $\langle \rho_{xy}\rangle ∼0.5$ after the CA event.

#### 2.1.1. Correlation Network

**Figure 4:**
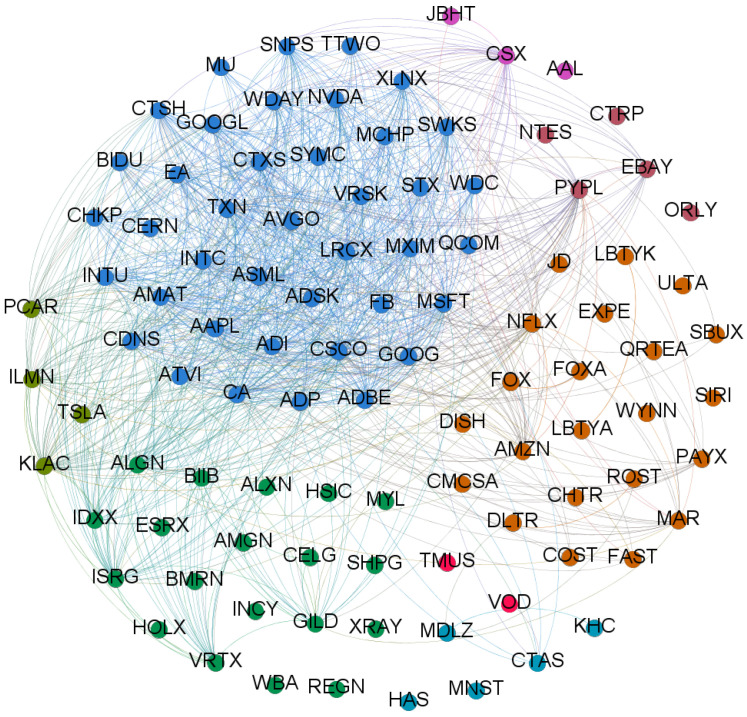
Graph displaying the NASDAQ-100 components grouped by industry. The list of industries is reported here below: • Technology    • Consumer Services • Health Care    • C. Non-Durables• Miscellaneous  • Capital Goods    • Transportation  • Public Utilities
In the graph, components are connected by an edge if their absolute cross-correlation over the whole time lapse is greater than 0.55. Colors follow the industry classification proposed by the NASDAQ according to the ICB system.

To highlight the structure of the stocks’ cross-correlations, we represent the correlation matrices as weighted networks. To this respect, related methodologies are presented in Section 4.2.2. In such networks, nodes represent stocks while edges represent significant correlations. In Figure 4, we show the NASDAQ-100 components network subdivided per industry according to a taxonomy, proposed by the NASDAQ, stemming from the Industry Classification Benchmark (ICB) system (Components’ list with classification available here: https://www.nasdaq.com/screening/company-list.aspx (July 30, 2018 5:35 pm)).

**Figure 5:**
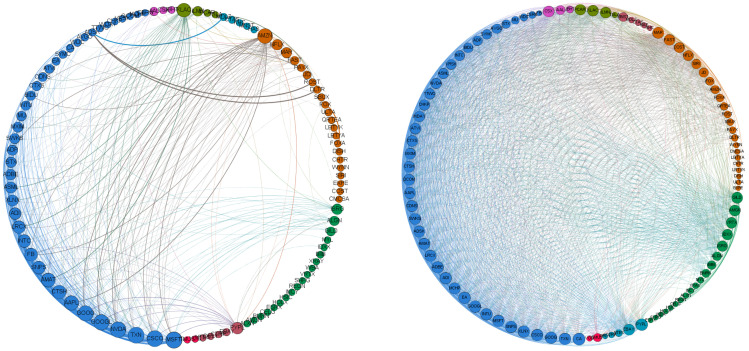
Correlation graphs with threshold, c=0.55. **Left panel:** Correlation graph *before* the CA event. **Right panel:** Correlation graph *after* the CA event. Nodes are ordered anticlockwise by industry (color) and node’s degree (size). Edge thickness varies according to absolute correlation. However, edge-thickness scales differ in the two panels to improve readability. Notice the increase in the number of edges, i.e., an increase in correlation, after CA.

In Figure 5, we show the correlation network among the NASDAQ-100 components before and after the CA event. We observe that the graph hints some structure of cross-industry correlation among specific assets before the CA scandal, whereas after the events of CA, correlations are denser among all the stocks and no clear cross-industry correlation structure appears.

**Figure 6:**
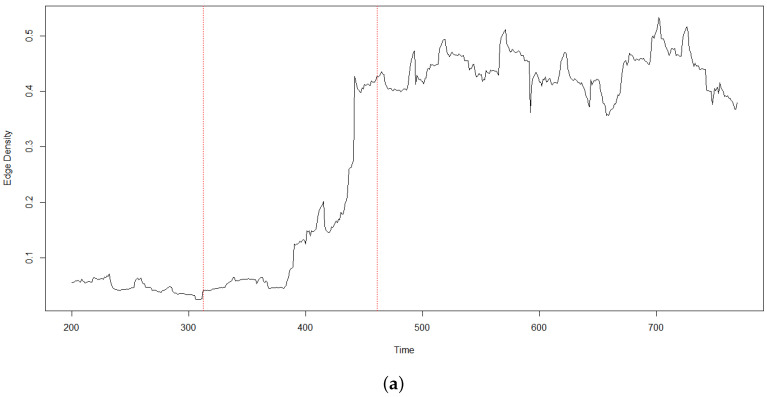

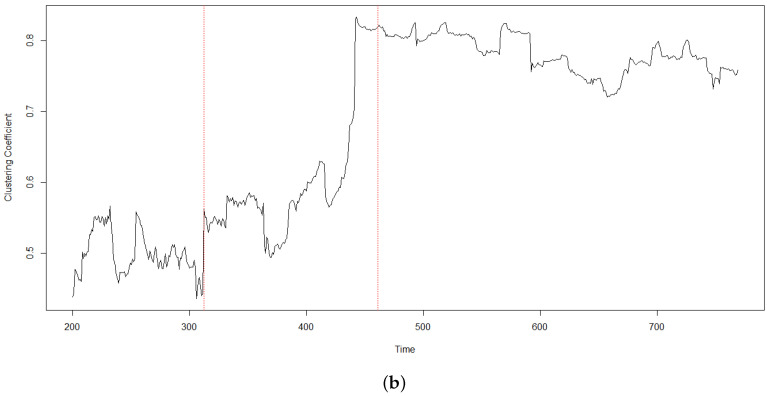
Edge density (**a**) and clustering coefficient (**b**) calculated with a moving window of 150 intraday observations. The dashed vertical red lines indicate respectively when CA enters and is fully within the moving window. Notice the sharp rise in both edge density and clustering coefficient.

Associated with a graph, there are several structural quantities, like edge density (measuring the fraction of edges of a graph respect all the possible edges) and clustering coefficient (measuring the local cliquishness [22]. In Figure 6 we show how, similarly to the cross-correlations of Figure 3, edge density and clustering coefficient also have a sharp rise corresponding to the CA event.

#### 2.1.2. Correlation Threshold Sensitivity

**Figure 7:**
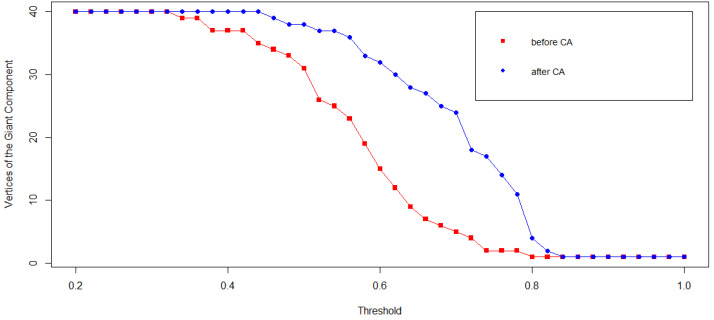
The sensitivity of the number of nodes belonging to the Giant Component *before* (**red** squares) and *after* (**blue** dots) CA. Notice that the size of the Giant Component grows after CA for all the meaningful correlation thresholds.

The sensitivity of the correlation network to different values of the correlation threshold *c* has been checked to look at the variation of the Giant Component for different values of *c* before and after the CA scandal. Figure 7 shows how the Giant Component consistently grows after CA for all the values of *c* between ∼0.35 and ∼0.80.

### 2.2. Mutual Information

Mutual Information (MI) is a measure of dependency for nonlinear time series [23]. It has been widely used in bio-informatics to cluster data while also taking into account finite size effect [24]. Generally measured in bits, it is a dimensionless quantity that can be interpreted as the reduction in uncertainty about one random variable given a perfect knowledge of the other. On the one hand, high MI reveals a large reduction in uncertainty. On the other hand, low MI indicates a great uncertainty on a random variable given the knowledge of the other; in particular, zero MI means that two random variables are independent.

Notice that it is possible to have non-zero MI even in presence of zero correlation: In fact, while MI is a distance between two probability distributions, correlation measures linear relationships between two random variables.

**Figure 8:**
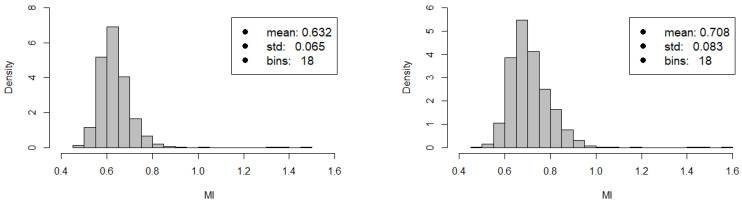
Distribution of the Mutual Information among the time series of the NASDAQ-100 stock market index. **Left panel:** Mutual Information among the time series *before* the CA event. **Right panel:** Mutual Information among the time series *after* the CA event. Mutual Information for each pair of stocks has been computed on two samples of 15-minute intraday stock returns consisting in 311 observations each. Notice that the average Mutual Information among the assets of the stock market experiences a ∼10% increase.

In Figure 8, we show how the histogram of the MI values varies across the stocks of the NASDAQ-100 before and after the CA event. After the CA event MI grows on average, i.e., the market becomes more predictable from the knowledge of a limited subset of stocks. Related methodologies are presented in Section 4.2.3.

**Figure 9:**
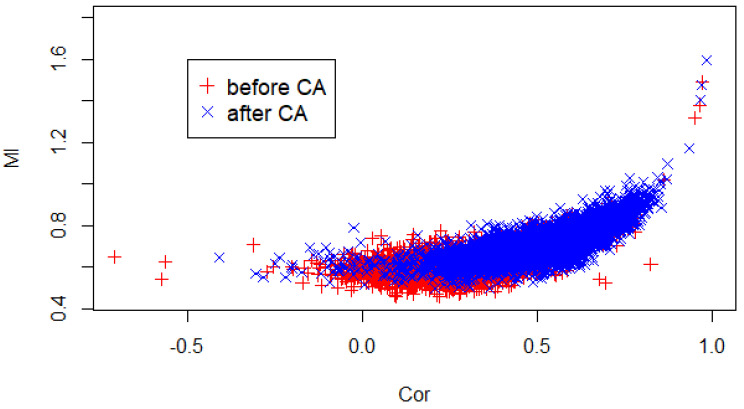
Scatter plot coupling Correlation and MI for every pair of stocks *before* (**red** pluses) and *after* (**blue** crosses) the events of CA. Notice that the all the points seem to follow a master curve.

It is also interesting to check the relation between MI and correlation, since it may allow us to spot possible methodological inconsistencies. Figure 9 shows the values of MI versus linear correlation before and after the CA event. MI is non-zero for zero correlations and increases for positive correlations; notice that the points of the scatter plot seem to follow a master curve. This is compatible with findings in similar cases [25]. However, we are not able to fully appreciate the characteristic U-shaped curve, given the absence of strongly negative correlations across the time series of the NASDAQ-100 components. In fact, the assets chosen by the NASDAQ are subject to common risk factors which mitigate the effect of possible sources of negative correlation.

## 3. Discussion

This work should be seen in the light of what has been done concerning the impact of news on financial markets. We have seen how studying a limited set of predictable announcements is frequent in the finance literature, while, in our opinion, taking into account a specific event is less common. In this paper, we have presented a case study concerning the impact of the notorious CA scandal not only on the FB stock, which directly suffered from a serious loss of reputation, but also on the whole market. In particular, we observe a sudden fall of the FB stock price and an increase in its volatility after the shock.

We observe that, in correspondence of the above-mentioned scandal, not only does the volatility of all the stocks increases on average, but both cross-correlation among the stocks and Mutual Information among their time series also increase. Hence, the system starts behaving like a whole, leading to an increase of the systemic risk due to possible cascading failures. In this situation, not only is it difficult to select low-risk investment portfolios, but the number of possible portfolios also decreases: In fact, many investors can unknowingly share the same investment strategy and they can all fail together in case of rare, unfavorable events. In such a situation, it is clear that an increase in cross-correlation leads to an underestimate of the risks and hence to a more fragile stock market.

It is worth highlighting that this case study presents at least two limitations. In the first place, the use of 15-minutes intraday data might be a source of bias [26]. However, we checked the consistency of our results using also daily data and they seem to confirm our findings, despite the poor number of observations within the time span considered. The second limitation is common to all the studies focusing on a single specific event in a quickly adapting environment. Unfortunately, it is not possible to rule out the presence of spurious events. For this reason, we decided to keep the time window as close as possible to the event considered.

Moreover, this case study leaves ample space for further research. First of all, the use of sophisticated econometric models might cast a light on the timing required by FB and by the market to react to the shock caused by the CA scandal. The way in which the increase in correlation and in MI spread across different sectors might also deserve a closer look. Eventually, a broader investigation might be performed considering the common reaction of different assets to a sufficiently large number of data scandals.

## 4. Materials and Methods

### 4.1. Data

For our analysis, we considered the list of equity securities included in the well-known stock market index NASDAQ-100. Our initial dataset contained 103 stock-price high-frequency time series of the NASDAQ-100 components. We removed three time series, namely BKNG, MELI, and FISV, because of issues related to data collection. The resulting dataset contains 100 time series and 779 observations, ranging from 1 March to 12 April 2018, with a 15-minute frequency. The aforementioned time span has been chosen in order to include CA-scandal early events. Data have been collected from Bloomberg.

### 4.2. Methods

We begin transforming stock-price time series into log-return time series. Let $p_{i}\left(t\right)$ be the price of a stock *i* at time *t*, the log-return, $r_{i}\left(t\right)$, of the stock *i* at time *t*, is defined as follows [27,28,29]:

(1) $$r_{i}\left(t\right)=\ln\left(\frac{p_{i}\left(t\right)}{p_{i}\left(t-1\right)}\right)$$

From the original sample of log-returns, we extract two subsamples consisting of 311 observations each. The first subsample, starting on 1 March 2018, contains the available observations before the break out of CA scandal, whereas the second subsample, starting on 19 March 2018, fully contains the early effects of CA scandal.

#### 4.2.1. Correlations

We proceed computing Pearson correlation pairwise for all the time series in our two subsamples. Pearson correlation, $\rho_{x,y}$, for a pair of time series, x(t) and y(t), is defined as [27,30]:

(2) $$\rho_{x,y}=\frac{\langle xy\rangle -\langle x\rangle \langle y\rangle }{\left(\langle x^{2}\rangle -{\langle x\rangle }^{2}\right)\left(\langle y^{2}\rangle -{\langle y\rangle }^{2}\right)}$$

where 〈〉 indicates the average over a fixed time window, i.e for a given time window [t,t+N] the average of a quantity *x* is $\langle x\rangle =N^{-1}\sum_{i=1}^{N}x_{t+i}$.

We originate two correlation matrices with our log-return time series before and after the CA event. Non-diagonal elements of each matrix may assume values between −1, maximum negative linear correlation, and 1, maximum positive linear correlation, whereas a value equal to 0 signals the absence of any linear correlation. In our case, non-diagonal elements report the correlation coefficient for every pair of stocks. Obviously, diagonal elements report the correlation of each stock with itself, thus their value is always equal to +1.

#### 4.2.2. Correlation Network

A further step is the creation of a weighted network. A weighted network is a triplet G=(V,E,w) where *V* is the set of vertexes (or nodes), E⊆V×V is the set of edges (or links), and the function *w* associates to each edge *e* its weight w(e). Given a correlation threshold *c*, we represent a correlation matrix *C* as a weighted network by identifying the NASDAQ-100 stocks as the set of nodes and associating to each element $|C_{ij}|>c$ and edge e=(i,j) with weight $w\left(e\right)=|C_{ij}|$. We call such a network associated with the correlation matrix *C* with threshold *c* the correlation network $G_{c}\left(C\right)$. This slightly differs from [28,29,31] with the intention of also considering large negative correlations.

#### 4.2.3. Mutual Information

Eventually, we also take into account MI. MI between two discrete random variables, *X* and *Y*, can be defined as follows [32,33]:

(3) $$I\left(X;Y\right)=\sum_{y\in Y}\sum_{x\in X}p\left(x,y\right)\log\left(\frac{p(x,y)}{p(x)p(y)}\right)$$

where p(x,y), p(x), and p(y) are respectively the joint and marginal probability distributions of X and Y.

In order to compute MI pairwise, we proceed with the discretization of our time series. We opted for a number of bins equal to $\sqrt{N}$, i.e $\sqrt{311}\approx 18$bins for each of the two subsamples.

## Figures and Tables

**Table 1:** The table reports a ranking of the 10 highest-volatility stocks in the NASDAQ-100 *before* and *after* Cambridge Analytica (CA). Volatility has been computed on two samples of 15-minute intraday returns consisting of 311 observations each.

Top-10 Highest Volatility Stocks						
Before CA				After CA		
Stock	Industry	SD(x)		Stock	Industry	SD(x)
DLTR	Consumer Services	0.01031		SHPG	Health Care	0.01013
ESRX	Health Care	0.00757		TSLA	Capital Goods	0.00857
JD	Consumer Services	0.00732		MU	Technology	0.00721
ADSK	Technology	0.00724		NFLX	Consumer Services	0.00704
MU	Technology	0.00703		NVDA	Technology	0.00684
ALXN	Health Care	0.00671		FB	Technology	0.00668
ROST	Consumer Services	0.00576		AMZN	Consumer Services	0.00640
WYNN	Consumer Services	0.00533		LRCX	Technology	0.00623
ULTA	Consumer Services	0.00496		AMAT	Technology	0.00564
LRCX	Technology	0.00477		INTC	Technology	0.00563

## Abbreviations

The following abbreviations are used in this manuscript:

FB:	Facebook
CA:	Cambridge Analytica
MI:	Mutual Information
